# What Is the True Standard of Mental Training?

**Published:** 1902-01

**Authors:** 


### Editorial.

### WHAT IS THE TRUE STANDARD OF MENTAL TRAINING?

THE question, "What constitutes a true standard of cultivation?" is, perhaps, one of the most difficult problems to decide. The individual who has reached the Ph.D. of our higher universities has no difficulty in answering this, and that without a moment's reflection, and his opinion will be in accord with the recognized standard of Caucasian civilization. It requires no argument to prove that this decision would not be acceptable to that older civilization, the oldest existing,—the Chinese,—and yet the difference in results obtained through the more modern and the older methods may not be clearly distinguishable; in fact, it may be said that the Chinese culture is in all respects the equal of that developed in more recent historical periods.

It is, therefore, very evident that it is not so much the character of training given the mind as it is that the mental forces should be kept in continued activity.

This naturally brings to the surface prominently, Is it essential that this mental training be confined to certain routine studies in order to reach a high standard of cultivation? The answer will be given always in accordance with the tastes and experiences of the individual. One will say that no man can be considered scholarly who has not a thorough familiarity with the classics. Another will regard Greek as unimportant, but that Latin is essential to mental training. The mathematician will give the languages a second place, regarding his favorite study as deserving a prominent position as a strengthener of the intellect. All of these combined will regard with contempt the man or woman deficient in these, and will unhesitatingly regard them as uneducated. It seems to the writer that this is a forced conception of the true meaning of mental development. While all of these studies occupy, and will probably ever continue to occupy, the most prominent place in the curriculum of the world, they are yet a part only of that educational training, and the majority must seek elsewhere and through altogether different methods to arrive at the same end,—the cultivation of the intellectual powers. The world is full of examples of great mental vigor accomplished without the aid of any university or college, and through entirely different channels of cultivation.

What constitutes a foundational education preparatory to the study of any subject? The colleges and universities vary in their several standards of entrance, but all are formed upon certain established lines, and these are prohibitory to the man trained in work of a different character. This will be right or wrong according to the point of view taken, but it certainly cannot be changed. While this is true, it does not follow that the standard is a correct one.

In some European countries, notably Germany, the man who wishes to be in one of the so-called learned professions—law, medicine, theology, etc.—must start from the Gymnasium; and the man who graduates from the Polytechnic school will never be able to reach these professions, no matter what may be his ambitions. It will hardly be contended that the school that prepares and graduates the great civil and mechanical engineers is not productive of an equal amount of mental growth as that developed under a different training in the Gymnasium. It seems that a more reasonable conception of the subject is desirable, and that the man of intellectual force should have recognition, whether this came to him through the universities or through mental development the result of constant contact with the world's activities.

It is constantly in evidence that this mistaken course warps the minds of those in responsible positions. They frequently assume conclusions based on recognized standards and not upon common sense methods of judgment.

The true test of a cultivated intellect lies not in the too rigid adherence to the minor details of an established curriculum, but in observing the mental capabilities of the individual to grasp the work of the world in its various departments of intellectual development, and the power to make this a part of the individual life. This will mean something more than is taught in the schools; but with that as an assured foundation, the mind will develop by the accretions of years and become fitted for all the higher responsibilities of life.

